# Bone marrow stem cells secretome accelerates simulated birth trauma-induced stress urinary incontinence recovery in rats

**DOI:** 10.18632/aging.202812

**Published:** 2021-03-31

**Authors:** Minghui Jiang, Jiahui Liu, Wenli Liu, Xiaoliang Zhu, Yasmeen Bano, Hongbing Liao, Haiyan Li, Hai-Hong Jiang

**Affiliations:** 1 Department of Urology, The First Affiliated Hospital of Wenzhou Medical University, Wenzhou 325000, China

**Keywords:** stress urinary incontinence, bone marrow stem cells, secretome, fibroblasts, JAK2/STAT4

## Abstract

Stress urinary incontinence (SUI) is defined as involuntary urine leakage during physical activities that increase the intra-abdominal pressure on the bladder. We studied bone marrow stem cell (BMSC) secretome-induced activation of anterior vaginal wall (AVW) fibroblasts and its ability to accelerate SUI recovery following vaginal distention (VD) in a rat model of birth trauma using BMSC-conditioned medium (BMSC-CM) and concentrated conditioned medium (CCM). BMSC-CM enhanced the proliferation, migration, and collagen synthesizing abilities of fibroblasts. Differentially expressed genes in BMSC-CM-induced fibroblasts were mainly enriched for cell adhesion, extracellular fibril organization and angiogenesis. Treatment with the JAK2 inhibitor AG490 reversed BMSC-CM-induced activation of the JAK2/STAT4 pathway. Periurethral injection with BMSC-CCM markedly enhanced the abdominal leak point pressure (LPP) in rats after VD. Histological analysis revealed increased numbers of fibroblasts, improved collagen fibers arrangement and elevated collagens content in the AVW of rats receiving BMSC-CCM. These findings suggest the BMSC secretome activates AVW fibroblasts and contributes to the functional and anatomic recovery of simulated birth trauma-induced SUI in rats.

## INTRODUCTION

Stress urinary incontinence (SUI) is defined as involuntary urine leakage while coughing, sneezing, or performing activities that increase the intra-abdominal pressure such as lifting and running. Hyperactivity of the urethra in SUI is attributed to an anatomical defect or sphincter deficiency [1]. Collagen, one of the primary components of extracellular matrix (ECM), is secreted by fibroblasts and assembled into fibers that form the structural scaffold of various connective tissues such as cartilage, bone, tendons, and ligaments. Patients with SUI have highly reduced collagen content in the anterior vaginal wall (AVW), pelvic fascia, and ligaments [2–4]. Several studies have associated birth trauma-induced injury to muscles, nerves and connective tissues, as well as postpartum recovery barriers, to the development of SUI [5–7]. Further, a mechanical stretch during vaginal birth can induce fibroblast apoptosis, reducing the production of ECM components, such as collagen, and causing SUI after vaginal birth [8].

Clinical trials [9, 10] and studies on animal models of simulated birth-trauma [11, 12] demonstrated the potential therapeutic functions of mesenchymal stem cells (MSCs) against SUI. MSCs exert therapeutic benefits via their self-renewal ability, multilineage differentiation, and potent paracrine/autocrine function. In addition, MSC secretome, containing numerous bioactive factors, participate in angiogenesis, tissue repair, immunomodulation, and anti-fibrotic effects [13–15]. The effects of MSC secretome on the recovered external urethral sphincter and paraurethral connective tissue, and improved leak point pressure (LPP), have been demonstrated in rat models [16, 17].

Previous studies have demonstrated that MSC secretome remarkably activates the fibroblasts both during skin wound healing and birth trauma-induced SUI [18, 19]. Therefore, we hypothesized that the human bone marrow stem cell (BMSC) secretome can be used to create a microenvironment that promotes the growth and survival of collagen-producing vaginal fibroblasts, thereby improving urinary continence in rats after simulated birth-trauma.

## RESULTS

### BMSC-CM facilitates the proliferation and migration abilities of fibroblasts

**Figure 1:**
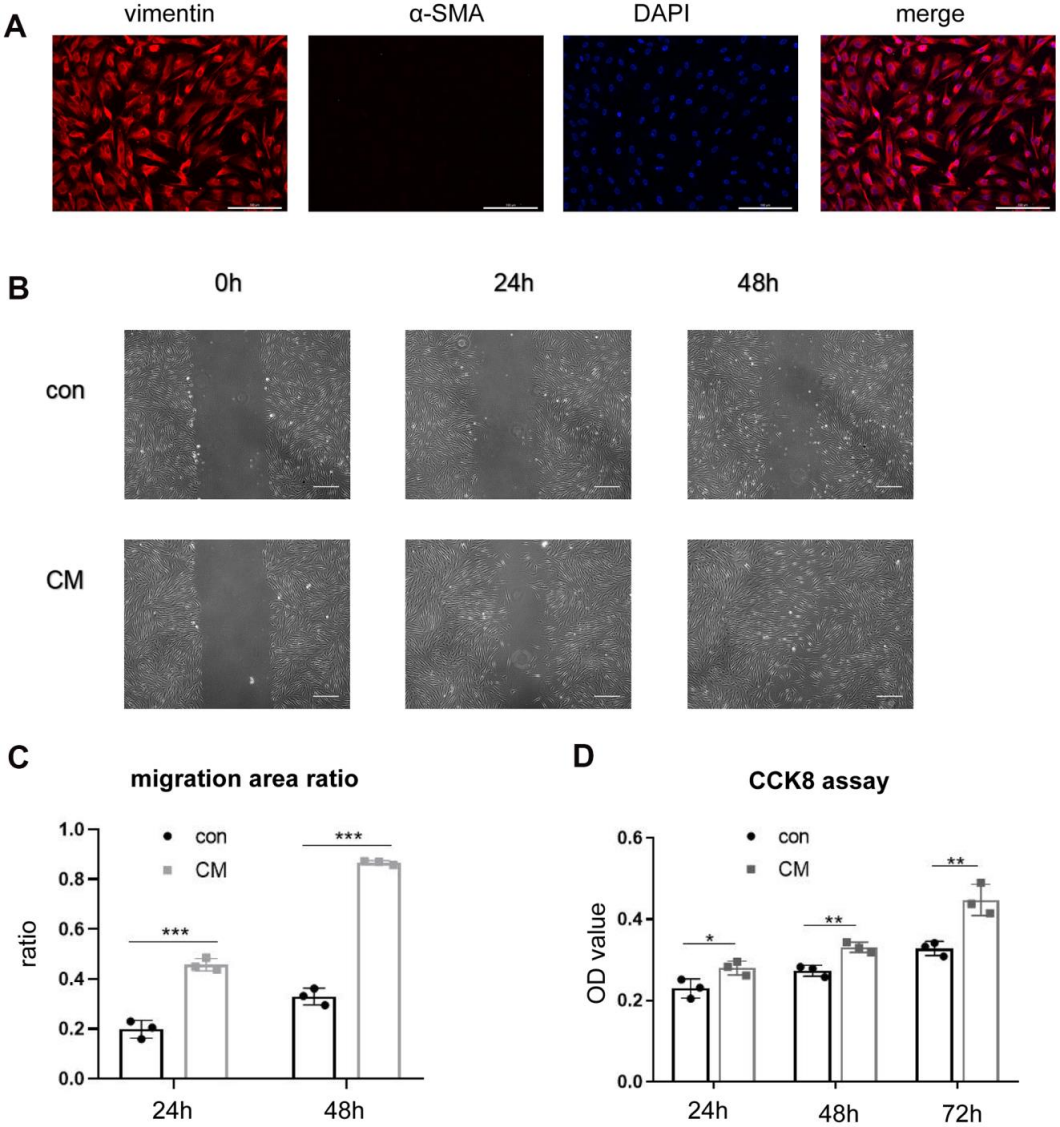
**BMSC-CM enhanced the proliferation and migration ability of fibroblasts *in vitro*.** (**A**) Positive staining of vimentin and negative staining of α-SMA in cultured fibroblasts by immunofluorescence (magnification, ×200). Scale bar = 200 μm. (**B**) Wound–scratch assay in fibroblasts treated with different media at 0 h, 24 h, 48 h (magnification, ×40). Scale bar = 400μm. (**C**) The percentage of migration area in different groups. (**D**) Cell number displayed as OD value of fibroblasts treated with different media in the CCK-8 assay. Data are shown as means ± standard deviation (SD). ^*^P < 0.05; ^**^P < 0.01; ^***^P < 0.001. BMSC-CM, bone marrow stem cell-conditioned medium; con, control medium treatment group; CM, BMSC-CM treatment group.

Fibroblasts were successfully isolated from the AVW tissue that exhibited vimentin positivity and α-SMA negativity (Figure 1A). A scratch-wound assay was performed to examine whether BMSC-CM exhibited biological effects on the migration of fibroblasts. As shown in Figure 1B, compared with the control medium (serum-free DMEM/F12), BMSC-CM had a more potent effect on cell migration after 24 h and 48 h of its addition. Figure 1C shows the quantification of the migration area. Furthermore, a CCK-8 assay was performed to study the effect of BMSC-CM on cell proliferation. As shown in Figure 1D, compared with the control medium group, the number of viable fibroblasts, assessed by determining the OD value, increased in the BMSC-CM group after 24 h, 48 h, and 72 h of its addition.

### BMSC-CM regulates collagen metabolism in fibroblasts

**Figure 2:**
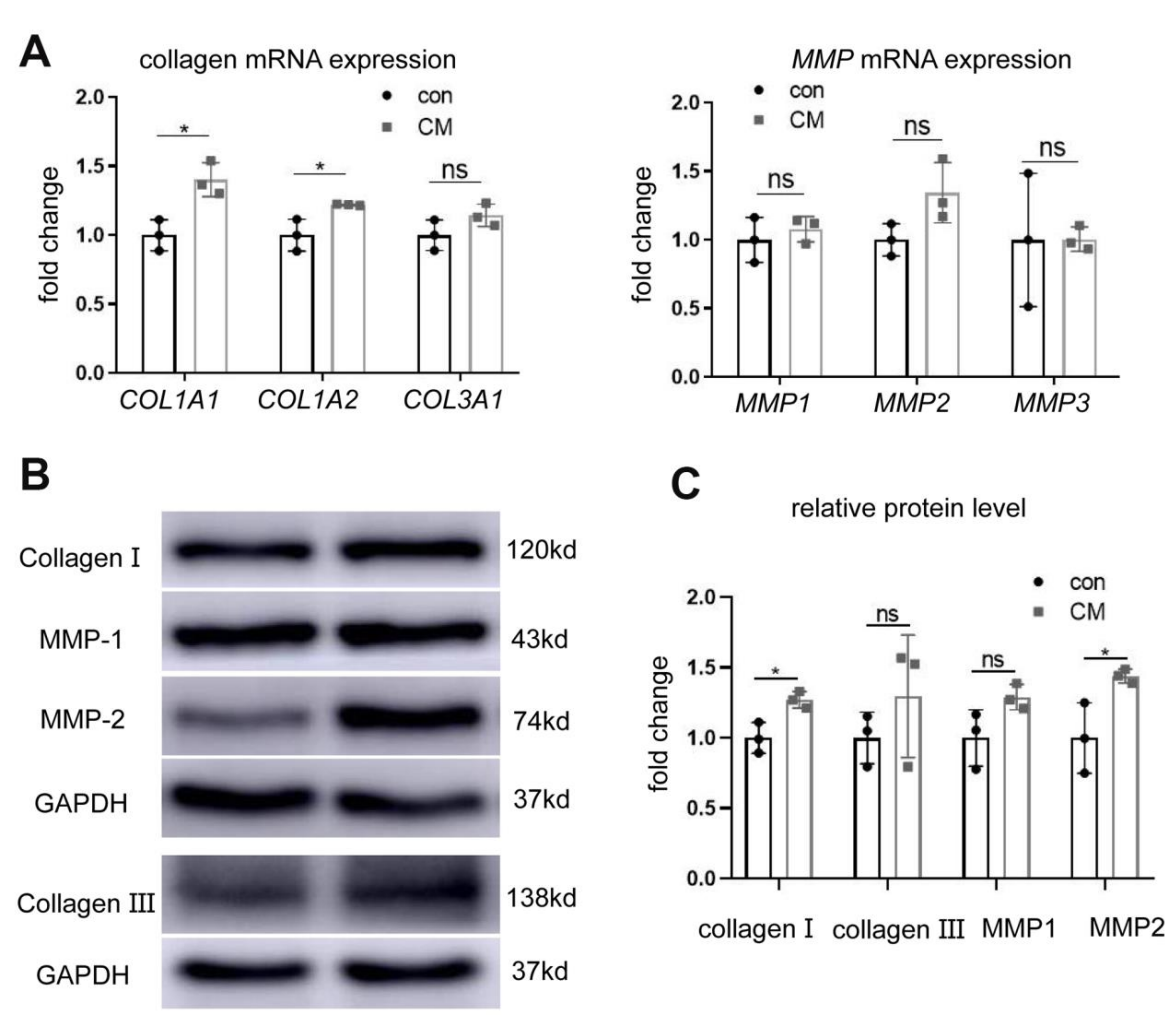
**BMSC-CM promoted collagen production in fibroblasts.** (**A**) RT-PCR showing relative mRNA expression of collagen type I alpha 1 chain (*COL1A1*), collagen type I alpha 2 chain (*COL1A2*), collagen type III alpha 1 chain (*COL3A1*), matrix metalloproteinases *MMP1*, *MMP2,* and *MMP3* in fibroblasts treated with different media (versus GAPDH mRNA). (**B**) Western blotting showing protein levels of collagen I, collagen III, MMP1, and MMP2 in the two groups of fibroblasts. (**C**) Relative collagen I, collagen III, MMP1 and MMP2 protein levels (versus GAPDH). Data are shown as means ± standard deviation (SD). ^*^P < 0.05; ^**^P < 0.01; ^***^P < 0.001. BMSC-CM, bone marrow stem cell-conditioned medium; con, control medium treatment group; CM, BMSC-CM treatment group.

To study the effects of BMSC-CM on collagen production and metabolism, the expression of collagen and its related regulators was measured at mRNA and protein levels. Reverse-transcription polymerase chain reaction (RT-PCR) revealed upregulated mRNA expression of collagen type I alpha 1 chain (*COL1A1*) and collagen type I alpha 2 chain (*COL1A2*) following BMSC-CM treatment (Figure 2A) rather than collagen type III alpha 1 chain (*COL3A1*) mRNA expression. Similarly, the protein expression of collagen I showed an upward trend (Figure 2B, 2C). However, the mRNA expression of matrix metalloproteinases (MMPs; *MMP1*, *MMP2,* and *MMP3*) remained unaltered between the two groups (Figure 2A). Western blotting experiments revealed no significant changes in MMP1 protein expression, whereas MMP2 protein expression was upregulated in the BMSC-CM group (Figure 2B, 2C).

### BMSC-CM alters the transcriptome profile of fibroblasts

**Figure 3:**
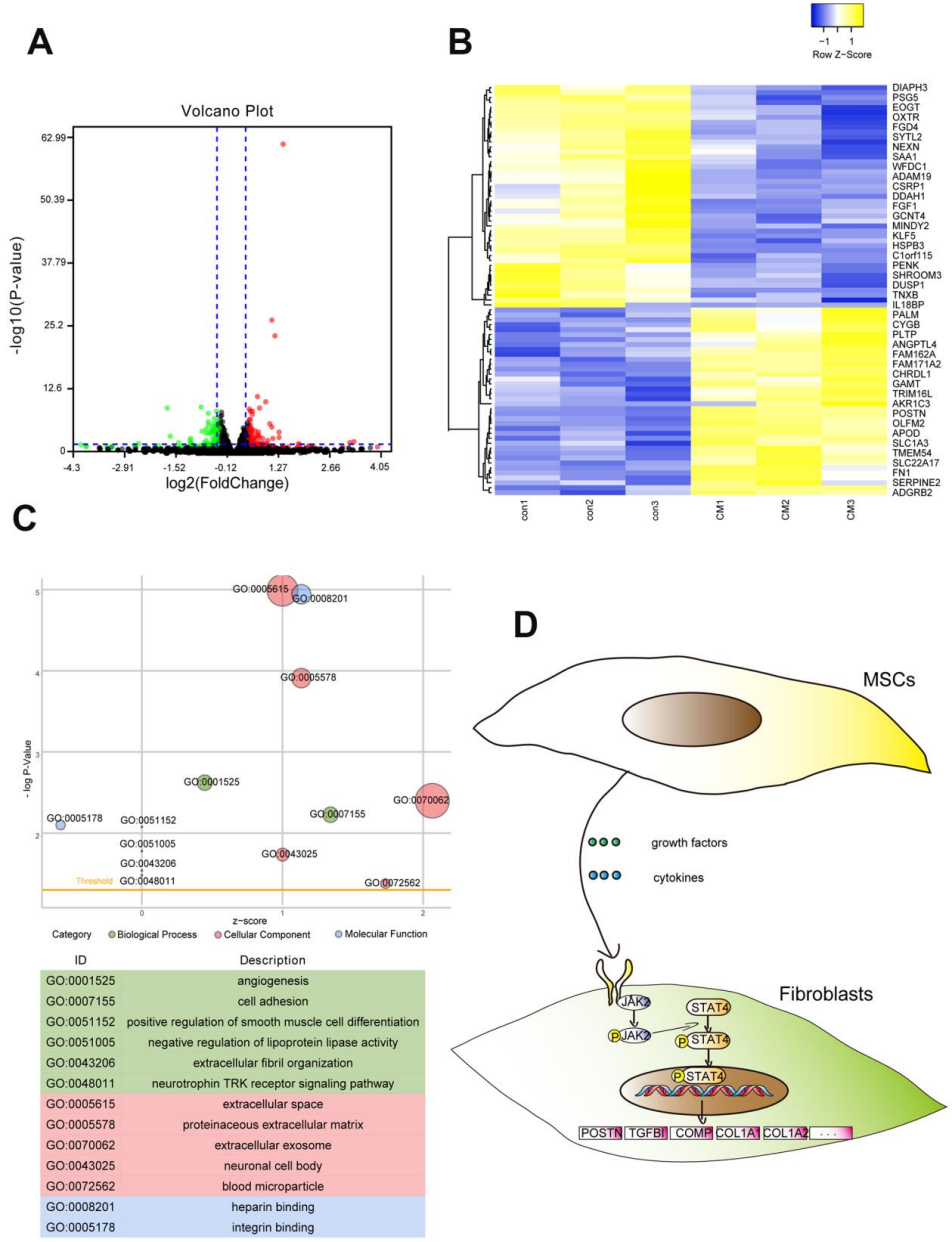
**RNA-seq revealed the whole genome expression changes in fibroblasts after treatment with BMSC-CM.** (**A**) Volcano plot showing DEGs of BMSC-CM group relative to the control group. The red plot indicates high expression, and the green plot indicates low expression. (**B**) The upper and lower panels showing heat maps of DEGs in fibroblasts. (**C**) The GO function analysis indicated DEGs to be enriched in biological processes, cellular components and molecular functions. The node size represented the number of genes enriched in the category. (**D**) Schematic diagram of possible signal transduction pathways according to RNA-seq results. DEGs, differentially expressed genes; BMSC-CM, bone marrow stem cell-conditioned medium; con, control medium treatment group; CM, BMSC-CM treatment group.

RNA-sequencing (RNA-seq) was performed to further determine the function of BMSC-CM in regulating the gene expression in fibroblasts. A total of 83 differentially expressed genes (DEGs) coding proteins were screened by RNA-seq (**|**fold change**|** > 1.3 and adjusted *p*-value < 0.05), including 38 upregulated and 45 downregulated genes (Figure 3A). Among them, *POSTN*, *COMP*, and *TGFBI* mRNAs showed a substantial increase (**|**fold change**|** > 2 and adjusted *p*-value < 0.05). The heat map revealed differential gene expression in fibroblasts treated with BMSC-CM compared with those treated with control medium (Figure 3B). The GO analysis (Figure 3C) showed that DEGs were mainly enriched in cell adhesion, extracellular fibril organization, and angiogenesis. PROMO is a database to predict potential transcription factors of genes according to experimental identification. The PROMO database showed that potential transcription factors regulating DEGs, such as *POSTN*, *COMP*, and *TGFBI*, indicated the involvement of signal transducer and activator of transcription 4 (STAT4) (Supplementary Table 1). Possible signal transduction pathways according to RNA-seq results are displayed in Figure 3D.

### BMSC-CM activates fibroblasts via the JAK2/STAT4 pathway

**Figure 4:**
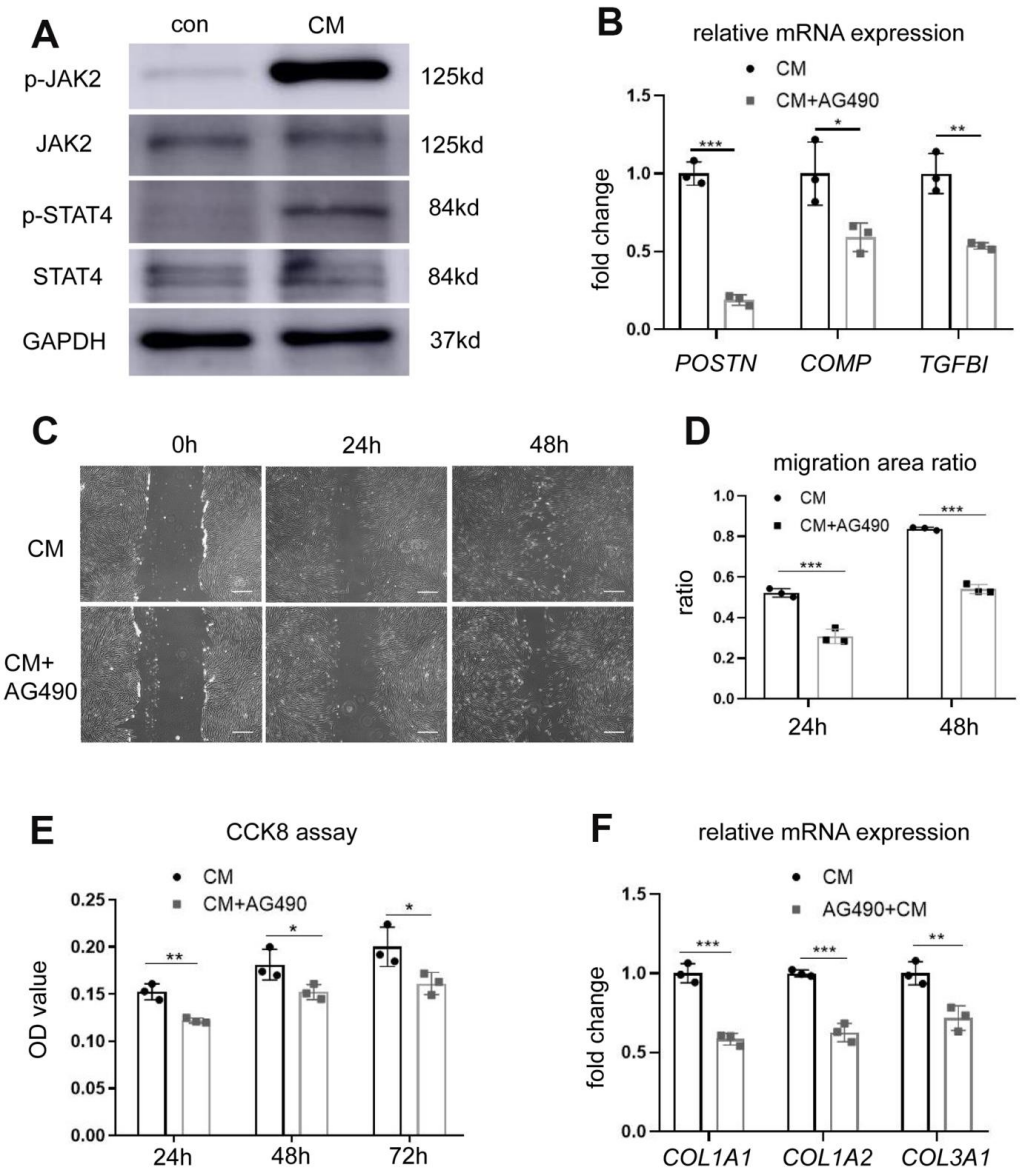
**BMSC-CM activated fibroblasts by activating the JAK2/STAT4 pathway.** (**A**) The expression of JAK2, p-JAK2, STAT4, and p-STAT4 in fibroblasts treated with different media was determined by western blotting. (**B**) The expression of three most significant DEGs (*POSTN*, *COMP*, and *TGFBI*) in fibroblasts treated with JAK2 inhibitor AG490 (10 μM). (**C**) Wound–scratch assay in fibroblasts treated with or without JAK2 inhibitor AG490 (10 μM) at 0 h, 24 h, and 48 h (magnification, ×40). Scale bar = 400 μm. (**D**) The percentage of migration area in different groups. (**E**) The CCK-8 assay showing reduced proliferation of fibroblasts treated with BMSC-CM after treatment with AG490. (**F**) Collagen synthesis in fibroblasts after AG490 treatment was determined by RT-PCR. Data are shown as means ± standard deviation (SD). ^*^P < 0.05; ^**^P < 0.01; ^***^P < 0.001. BMSC-CM, bone marrow stem cell-conditioned medium; con, control medium treatment group; CM, BMSC-CM treatment group.

To investigate the alterations in signal transduction pathways associated with enhanced proliferation and migration ability of fibroblasts following BMSC-CM stimulation, we studied the involvement of STAT4 and its upstream JAK2 molecules in these events. Western blotting revealed elevated levels of p-JAK2 and p-STAT4 in fibroblasts cultured with BMSC-CM compared with those cultured in the control medium (Figure 4A). Treatment with 10 μM JAK2 inhibitor AG490 decreased the mRNA expression of *POSTN*, *COMP,* and *TGFBI* in BMSC-CM-cultured fibroblasts (Figure 4B), demonstrating the involvement of the JAK2/STAT4 pathway in BMSC-CM-cultured fibroblasts. We further checked whether the inhibition of JAK2 suppressed the proliferation and migration of fibroblasts cultured in BMSC-CM. As shown in Figure 4C, 4D, treatment with AG490 suppressed the cell migration ability of fibroblasts in the BMSC-CM culture. Similarly, AG490 remarkably inhibited the proliferation ability of fibroblasts cultured in BMSC-CM (Figure 4E). Moreover, AG490 decreased the mRNA levels of *COL1A1* and *COL1A2* in BMSC-CM-cultured fibroblasts (Figure 4F). These results concluded that BMSC-CM activated fibroblasts via the JAK2/STAT4 pathway.

### BMSC-CCM enhances leak point pressures in rats with vaginal-distention

**Figure 5:**
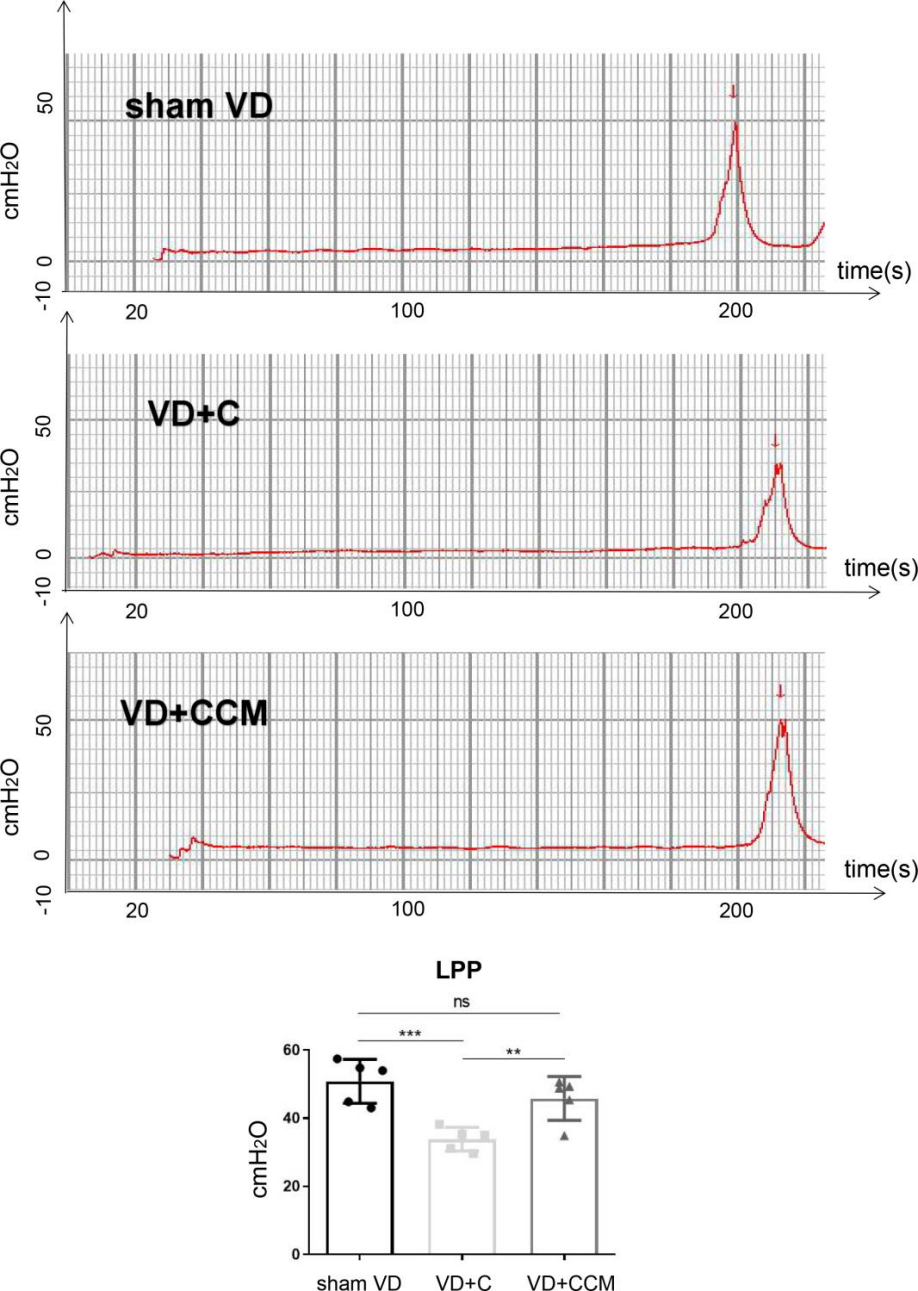
**BMSC-CCM treatment enhanced LPP of rats under VD.** Measurement and quantification of LPP in three rat groups. Data are shown as means ± standard deviation (SD). ^*^P < 0.05; ^**^P < 0.01; ^***^P < 0.001. BMSC-CCM, bone marrow stem cell-concentrated conditioned medium; LPP, leak point pressure; VD, vaginal distention; red arrow: urinary leakage; VD + C, VD + control medium group; VD + CCM, VD + BMSC-CCM group.

We next studied the effect of BMSC-CCM on the urethral function of simulated birth trauma in a rat model. The urethral function, represented by LPP (Figure 5), decreased in rats with VD as compared with that in the sham VD group (50.82 ± 6.45 cmH_2_O vs. 33.84 ± 3.46 cmH_2_O), suggesting that VD models were successfully established. However, treatment with concentrated conditioned medium (CCM) reversed the decreased LPP in the VD group (45.80 ± 6.42 cmH_2_O vs. 33.84 ± 3.46 cmH_2_O). In addition, no difference was found in LPP between the sham VD and VD + CCM groups, revealing that BMSC-CM facilitated urethral functional recovery. Furthermore, no unstable contraction waves were observed in the three groups.

### BMSC-CCM accelerates the survival of fibroblasts and regeneration of collagen fibers in rat anterior vaginal wall after VD

**Figure 6:**
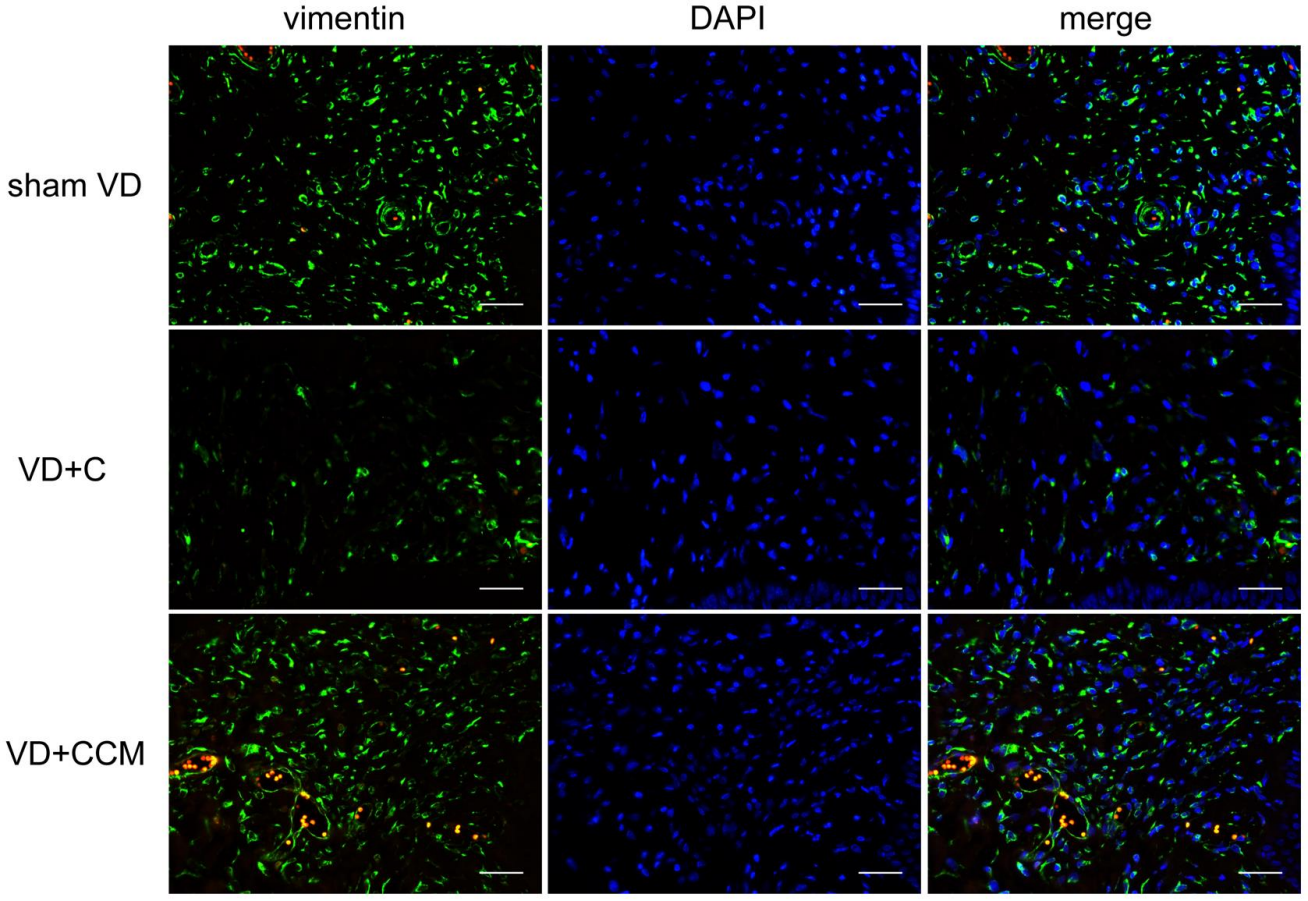
**BMSC-CCM treatment accelerated the survival of fibroblasts in AVW.** The number of vaginal wall fibroblasts in rats was assessed by observing vimentin-positive cells (magnification, ×400). Scale bar = 40 μm. BMSC-CCM, bone marrow stem cell-concentrated conditioned medium; VD, vaginal distention; VD + C, VD + control medium group; VD + CCM, VD + BMSC-CCM group.

**Figure 7:**
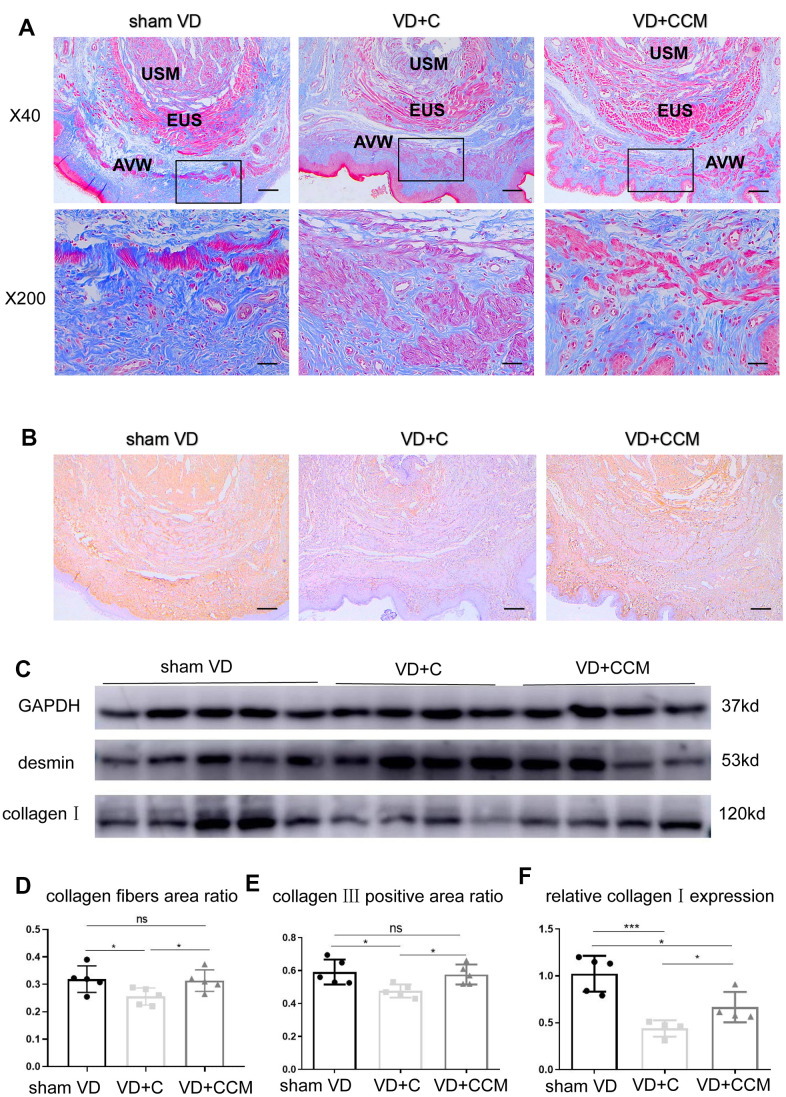
**BMSC-CCM treatment promoted collagen content in the middle urethra and adjacent AVW tissues of VD rats.** (**A**, **D**) Masson’s tricolor staining of the middle urethra and adjacent AVW tissues collected from rats. Collagen fibers were stained blue. The images were magnified 40× (scale bar = 200 μm) and 200× (scale bar = 40 μm). (**B**, **E**) IHC showing collagen III (stained brownish yellow) expression in tissues (scale bar = 200 μm). (**C**, **F**) The expression of desmin and collagen I was determined by western blotting, and the relative collagen I expression (vs. GAPDH) was analyzed. Data are shown as the means ± standard deviation (SD). ^*^P < 0.05; ^**^P < 0.01; ^***^P < 0.001. BMSC-CCM, bone marrow stem cell-concentrated conditioned medium; VD, vaginal distention; EUS, external urethral sphincter; AVW, anterior vaginal wall; USM, urethral smooth muscle; VD + C, VD + control medium group; VD + CCM, VD + BMSC-CCM group.

Increased LLP could be explained by the presence of fibroblasts and the secreted collagen fibers. The number of vaginal wall fibroblasts assessed by vimentin-positive cells decreased in rats treated with VD + control medium compared with that in the sham VD group. However, rats treated with VD + BMSC-CCM did not show such an effect (Figure 6), suggesting that BMSC-CCM accelerated the VD-inhibited survival of fibroblasts. Collagen fibers were largely observed within the submucosa and serosa of the vaginal wall, whereas only a small part was located within the urethral smooth muscles (USM) between the external urethral sphincter (EUS) and urothelium (Figure 7A). The number of collagen fibers in the VD group was less than that in the sham VD group, especially in the serosa. BMSC-CCM treatment ameliorated VD-induced reduction in the density of collagen fibers. Furthermore, there were more clumped collagen fibers in the VD + CCM group, suggesting that CCM facilitated the production of newborn collagen fibers. The quantitative analysis of collagen fibers (Figure 7D) showed that the total content of collagen fibers in the VD + CCM group did not show any difference as compared with the sham VD group. However, it was more than that in the VD group. Immunohistochemistry and western blotting revealed changes in protein levels in the urethra and anterior vaginal wall. Compared with the VD group, tissues from the VD + CCM group had increased collagen III -positive area ratio but no major difference as compared with the sham VD group (Figure 7B, 7E). Similarly, rats in the VD + CCM group had a higher collagen I expression in the urethra and AVW compared with the VD group; however, this was still lower than that in the sham VD group (Figure 7C, 7F). These results showed that BMSC-CCM accelerated the production of collagen fibers in the vaginal wall.

## DISCUSSION

Neuromuscular damage and lack of pelvic connective tissue support, during and after vaginal birth, have been implicated in the etiology of SUI. Several studies have suggested that the development of birth trauma-induced SUI is accompanied by fibroblast dysfunction and reduced collagen content [20]. The mechanical stretch induced by vaginal childbirth increases the intracellular reactive oxygen species (ROS) levels and cell apoptotic rate and reduces collagen type I alpha1 chain expression in human parametrial ligament fibroblasts [21]. Furthermore, a transcriptomics study revealed that the mechanical stretch disrupted fibroblast integrity and identified four mechanoresponsive genes regulating cytoskeleton remodeling and interaction with the ECM [22]. This study suggested that improved fibroblast microenvironment after vaginal birth may suppress the SUI process.

Recent preclinical and clinical studies have revealed the potential therapeutic function of MSCs in SUI [23, 24]. It was previously believed that MSCs exerted therapeutic benefits via their self-renewal ability and multilineage differentiation. However, several studies have now indicated that the survival time of MSCs, after implantation, is extremely short to exert a potent impact ^1^. The potential effects of MSCs could be attributed to MSC secretome that contains numerous bioactive factors [13, 15, 25]. Mass spectrometry-based proteomics analysis and enzyme-linked immunosorbent assay (ELISA) demonstrated that MSCs secrete a wide range of growth factors (GFs) and cytokines [26–28] that that integrate cell signal transduction pathways that regulate multiple cell behaviors.

MSC secretome exerts variable effects on fibroblasts depending on fibroblast source and different environmental conditions. For example, BMSC-CM, containing the complete BMSC secretome inhibited the viability of scar-derived fibroblasts [29]. In contrast, BMSC-CM increased the activation of diabetic human dermal [30] and L929 dermal fibroblast cell lines [31]. Consistently, our study demonstrated that bioactive factors in BMSC-CM facilitated the proliferation and migration of AVW fibroblasts *in vitro*. Several groups have reported the effects of bioactive factors on fibroblasts obtained from different sources. For instance, Lee et al. reported reduced migration of human dermal fibroblasts by neutralizing antibodies against vascular endothelial growth factor (VEGF) and basic fibroblast growth factor (bFGF) in adipose-derived stem cells conditioned medium [32]. Furthermore, inactivation of transforming growth factor-beta 1 (TGF-β1), present in amniotic fluid-derived stem cells conditioned medium, by specific inhibitor SB431542 strongly restrained human dermal fibroblast migration [33].

Despite displaying similar phenotypic changes following MSC-CM treatment, fibroblasts substantially differ in their gene expression patterns; these differences are attributable to fibroblast source, age of the host, and the culture medium used for propagating fibroblasts. Our RNA-seq analysis revealed the effects of BMSC-CM on vaginal wall fibroblasts isolated from adults with incontinence stress. The Gene Ontology (GO) enrichment analysis showed that DEGs were largely associated with cell adhesion, ECM organization, and angiogenesis, consistent with the enhanced proliferation, migration, and collagen synthesis abilities of BMSC-CM-treated fibroblasts.

We further studied the effect of the BMSC-CM-activated JAK2/STAT4 signal transduction pathway on fibroblasts. JAK2 is activated by several GFs and cytokines, such as IL-6, TNF, EGF, and PDGF. STAT4 is predominantly phosphorylated by activated JAK2 (p-JAK2) at tyrosine 693 and translocated to the nucleus as a dimer [34]. It binds to the promoter region of specific genes and participates in cell proliferation, apoptosis, and differentiation. We observed that BMSC-CM increased both p-JAK2 and p-STAT4 levels in the fibroblasts, which could be attributed to the binding of GFs and cytokines to membrane receptors. Treatment with JAK2 inhibitor AG490 reversed BMSC-CM-induced proliferation, migration, and collagen production in the fibroblasts. Above results showed that JAK2 plays a critical role in fibroblasts activated by BMSC-CM. RNA-seq results showed STAT4 as the transcription factor regulating DEGs. Whether STAT4 is the only downstream factor of JAK2 remains to be verified.

Various animal models of simulated birth trauma have been used to mimic the injuries causing SUI [35, 36]. Differences in the modeling method, researcher’s manipulations, measurement time, and animal species, affect the validity of models. We used animal models with VD and no pudendal nerve injury to minimize the effect of neuromuscular function on urinary continence. Compared with the sham VD group, LPP in the VD group decreased suggesting that damage to the urethra and related connective tissues can effectively induce urinary incontinence. After VD, local injection of BMSC-CCM ameliorated LPP indicating that its bioactive factors improved the recovery from simulated birth trauma-induced SUI. However, it remains unclear if these effects are applicable to EUS, USM, nerve, connective tissue or other incontinence factors.

Restoration of urethral function after VD via connective tissue regeneration and repair involves 1 to 4 weeks [37, 38]. We have previously shown that LPP and EUS electromyogram (EMG) of rats recovered 3 weeks after VD [39], indicating that tissue self-regeneration occurred within 3 weeks. In addition, we observed connective tissue regeneration at 2 weeks. Collagen I and collagen III, also known as interstitial collagens, are major components of the vaginal wall connective tissue. Collagen I confers strength to tissues, whereas collagen III contributes to elasticity. Several studies have shown decreased content and abnormal morphology of collagen I and collagen III in the AVW in patients with pelvic organ prolapse (POP) and SUI [2, 3, 40]; however, opposite results have also been reported [41]. The increased collagen content of the vaginal wall is partially responsible for enhanced LPP and improved incontinence [42]. Our study demonstrated that BMSC-CM promoted collagen I synthesis at both mRNA and protein levels *in vitro*. The collagen content is regulated by both synthesis and degradation. Patients with SUI and POP show upregulated MMPs—a family of ECM degradation-related proteins, and downregulated tissue inhibitors of metalloproteinases (TIMPs) [4, 43]. However, we did not observe differences in the expression of MMPs after BMSC-CM treatment, except for MMP2 protein. MMP1 primarily cleaves interstitial collagen, whereas MMP2 breaks down cleaved collagen fragments into amino acids. We speculate that individually elevated MMP2 levels had a weak effect on collagen metabolism. *In vivo*, BMSC-CCM increased the number of fibroblasts, the content of collagen fibers, and the expression of collagen protein in the AVW, consistent with the *in vitro* enhanced proliferation ability, accelerated migration rate, and increased collagen I following BMSC-CM treatment. In addition, collagen III in the vaginal wall increased after BMSC-CCM treatment; however, collagen III mRNA and protein expression remained unaltered, which could be attributed to post-transcriptional modifications and complex MMPs/TIMPs regulations. Based on our results, we conclude that VD-induced local mechanical strain inhibits the survival, migration, and collagen synthesizing abilities of fibroblasts, whereas BMSC-CCM bioactive factors facilitate the recovery of fibroblast activities.

Our study had several shortcomings due to limited laboratory conditions. Simulated birth trauma-induced alterations in fibroblasts could not be assessed; therefore, the effect of BMSC-CM on potentially defective fibroblasts remained unclear. To overcome this limitation, we examined the number of AVW fibroblasts in rats under different interventions. Next, we did not evaluate LPP at the time of VD. However, we assessed the LPP of sham VD to amend this limitation. Furthermore, we could not eliminate other factors, such as EUS, USM, and nerve reinnervation that affect the urethral function. Therefore, the contribution of increased collagen fibers and alterations in related pathways to improved incontinence remains uncertain.

In conclusion, our study demonstrates that BMSC-CM facilitates the proliferation, migration, and collagen production in AVW fibroblasts via the JAK2/STAT4 pathway to improve urinary incontinence and accelerate the regeneration of AVW collagen fibers in an SUI rat model following simulated birth trauma.

## MATERIALS AND METHODS

### Cell culture and identification

Fibroblasts were extracted from the AVW of a patient with SUI and vaginal prolapse using the method described previously. The tissue was shredded into small pieces, digested with 0.5% Type I collagenase for 60 min and further digested with 0.25% trypsin for 10 min. The resulting product was filtered through a 70-μm strainer to remove undigested fragments and subsequently centrifuged. The cell pellet collected was resuspended and seeded in Dulbecco's modified Eagle's media (DMEM, Gibco, USA), containing 20% fetal bovine serum (FBS, Gibco, USA) and 1% penicillin-streptomycin, and incubated at 37° C with 5% CO_2_. When reaching a 90% confluence, the cells were sub-cultured and the culture serum concentration was reduced to 10%. The cells between the third and the eighth culture passages were used for subsequent experiments. The cells at passage 3 were identified as fibroblasts by morphological observation and immunofluorescence (IF) staining. For IF, the cells were fixed with 4% paraformaldehyde, permeabilized with 0.25% Triton X-100, blocked with 5% BSA (BioFroxx, Germany), and incubated with mouse anti-vimentin (1:200, Proteintech, 60330-1-lg, USA) and rabbit anti-α SMA (1:200, Affinity Biosciences, AF1032, USA) antibodies overnight at 4° C. DyLightTM 594-labeled anti-mouse IgG (1:200, BoYun Biotech Co.) and DyLightTM 488-labeled anti-rabbit IgG (1:200, BoYun Biotech Co.) were used as secondary antibodies for 60 min at 37° C. Next, 4,6-diamino-2-phenylindole (DAPI; Solarbio, S2110, China) was used to stain the nuclei. The slides were observed and images were acquired using a fluorescence microscope (Leica, DM4000B, Germany).

The human BMSCs were purchased from Shanghai Zhongqiao Xinzhou Biotechnology Co. (ZQ0308, China), maintained in DMEM, supplemented with 10% FBS and 1% penicillin-streptomycin in a humidified atmosphere with 5% CO_2_. Cell passages were performed every 2 days at 1:3 dilution.

### Preparation of BMSC-CM

BMSCs were cultured and expanded in 10 cm dishes. When cells grew to 70% to 80% confluence, the medium was replaced with serum-free DMEM/F12 (Gibco, USA). After 48 h, cell supernatant was collected, centrifuged at 2000 g for 5 min, and finally filtered using a sterile 0.22-um filter (Millipore, USA) to obtain BMSC-CM. BMSC-CM was mixed with serum-free DMEM/F12 to prepare 50% BMSC-CM for subsequent experiments. For *in vivo* experiments, CM was further concentrated (10×) by ultrafiltration using centrifugal filter units with a 3-kDa cutoff (Millipore, USA) to obtain CCM.

### CCK-8 assays

The proliferation ability of the cells was determined by a Cell Counting Kit-8 (CCK-8; MultiSciences Biotech Co., China) in triplicate. Fibroblasts (3 × 10^3^ cells/well) were plated overnight in 96-well plates in DMEM with 0.5% FBS to adhere, and the medium was replaced with serum-free DMEM/F12 or 50% BMSC-CM. The proliferation of fibroblasts was measured every 24 h. 10 μl of CCK-8 solution was added to each well and incubated for 3 h. The absorbance was detected at 450 nm using an absorbance microplate reader (Molecular Devices, SpectraMax Plus 384, USA).

### Wound-scratch assay/cell migration assay

For cell migration assay, the cells were cultured in a 6-well plate at a density of 2 × 10^5^ cells/well in triplicate. The medium was replaced overnight with serum-free DMEM/F12 when the cells formed a confluent monolayer. Next, the wells were scratched with a 200 μL sterile pipette tip to create a linear gap in the confluent cell monolayer. After washing thrice, serum-free DMEM/F12 or 50% BMSC-CM was added to the respective wells. To evaluate the migration area, four randomly selected points were marked on each well. Images of the migration areas were acquired daily using an inverted microscope (Olympus, CKX53, Japan).

### Western blotting

To detect the protein expression after BMSC-CM treatment, fibroblasts (3 × 10^6^ cells) were seeded in 100 mm dishes in triplicate. Whole proteins (three from serum-free DMEM/F12-treated cells and the other three from BMSC-CM-treated cells) were extracted and their concentrations were determined using a bicinchoninic acid (BCA) protein assay kit (Thermo Fisher Scientific, USA). The whole proteins from each sample were separated on 10% SDS-PAGE gels and blotted onto polyvinylidene difluoride (PVDF) membranes (Millipore, IPVH00010, USA). After blocking with 5% BSA, the membranes were incubated overnight at 4° C with antibodies against GAPDH (1:1000, Affinity Biosciences, AF7021, USA), collagen I (1:1000, Affinity Biosciences, AF7001, USA), collagen III (1:1000, Abcam, ab7778, USA), MMP1 (1:1000, ABclonal, A1191, USA), and MMP2 (1:1000, Proteintech, 10373-2-AP, USA). After washing thrice with TBST, the membranes were incubated with horseradish peroxidase (HRP)-conjugated secondary antibodies. The protein bands were detected by chemiluminescence. The intensity of protein bands was measured using Image-J.

### Reverse transcription-polymerase chain reaction

To assess the effect of BMSC-CM on gene expression, fibroblasts (3 × 10^6^ cells) were cultured in 60 mm dishes in triplicate. Total RNA was extracted using the TRIzol reagent (Ambion, Life Technologies, USA) according to the manufacturer's protocol, and quantified by spectrophotometry (DeNovix, DS-11, USA). Subsequently, cDNA was obtained using the RevertAid First Strand cDNA Synthesis Kit (Thermo Fisher Scientific, USA). Next, cDNA templates were amplified using an SYBR Green Master Mix Kit (ABI, USA) and using the primers shown in Table 1. Three replicates of every sample were used and the procedure was set under the following conditions: pre-denaturation at 95° C for 2 min, followed by 40 cycles of denaturation at 95° C for 15 s, annealing at 55° C for 15 s, and elongation at 722103 for 45 s in a PCR instrument (Thermo Fisher Scientific, ABI QuantStudio 5, USA). The mRNA levels were calculated by the 2^–ΔΔCt^ method and normalized to that of GAPDH.

**Table 1:** Primer sequences used in RT-PCR.

**Gene**	**Primer sequence (5’-3’)**
GAPDH	F: GGAGCCAAAAGGGTCATCATCTC R: AGGCATTGCTGATGATCTTGAGG
COL1A1	F: TGGCAAAGAAGGCGGCAAAGG R: AGGAGCACCAGCAGGACCATC
COL1A2	F: TTCCAAGGAAATGGCTACCCAAC R: CTTTTTCAGGTTGCCAGTCTCCT
COL3A1	F: TGGATCTCCAGGATACCAAGGAC R: CGGGTCTACCTGATTCTCCATCT
MMP1	F: CAAATGGGCTTGAAGCTGCTTAC R: GGGTATCCGTGTAGCACATTCTG
MMP2	F: CGGCGGTCACAGCTACTTCTTC R: GCAGCCTAGCCAGTCGGATTTG
MMP3	F: CAACTGTGATCCTGCTTTGTCCT R: TGCAATTCAGGTTCAAGCTTCCT
POSTN TGFBI COMP	F: GGAAAACAGCAAACCACCTTCAC R: TTATTCACAGGTGCCAGCAAAGT F: AGGAATTTGCTTCGGAACCACAT R: GCTGTTCTCAATGCAGAGGCTAT F: GGAAGCAGATGGAGCAAACGTAT R:CTGGGACTCTGTGTCTCCTGTAT

### RNA-seq and analysis

The RNA samples used for RNA-seq were extracted using the above method (three samples from serum-free DMEM/F12-treated fibroblasts and the other three from BMSC-CM-treated fibroblasts). The RNA sample quality was assessed using the RNA Nano 6000 Assay Kit of the Bioanalyzer 2100 system (Agilent Technologies, CA, USA), and 1 μg of RNA per qualified sample was used to construct cDNA libraries following the manufacturer’s recommendations for the NEBNext® Ultra™ RNA Library Prep Kit for Illumina® (#E7530L, NEB, USA). The cDNA libraries were accurately quantified by the StepOnePlus™ qPCR System (library valid concentration > 10 nM) and sequenced on an Illumina HiSeq platform with 150 bp paired end reads. For the analysis, the original data (Raw data) were filtered using Perl script to obtain clean data for subsequent analyses. The clean data were mapped to the Ensembl database using HISAT2 (v2.1.0). The Integrative Genomics Viewer (IGV) was used to view the mapping results as heat maps, histograms, and scatter plots. The mapping reads of each gene and for each sample were counted using HTSeq (v0.6.0), and Fragments Per Kilobase of transcript per Million mapped reads (FPKM) were subsequently calculated to estimate the gene expression of each sample. The differential gene expression between the two samples was analyzed using DESeq2. Genes with an adjusted *p*-value ≤ 0.05 and fold change ≥ 1.3 were identified as DEGs. Gene ontology (GO) enrichment analyses were performed to study the functions of DEGs using the Database for Annotation, Visualization, and Integrated Discovery (DAVID) online database (https://david.ncifcrf.gov), whereas protein-protein interaction (PPI) networks were constructed using STRING (https://string-db.org). The PROMO online database (http://alggen.lsi.upc.es/cgi-bin/promo_v3/promo/promoinit.cgi?dirDB=TF_8.3) was used to predict transcription factors that regulate the expression of DEGs within a dissimilarity margin ≤ 5%.

### Detection and verification of the JAK2/STAT4 pathway

Alterations of pathways in BMSC-CM-cultured fibroblasts were detected by western blotting. The above-mentioned steps were performed and antibodies against JAK2 (1:1000, CST, 3230S, USA), p-JAK2 (1:1000, CST, 4406S, USA), STAT4 (1:1000, HuaBio, ET1701-42, China) and p-STAT4 (1:1000, Affinity Biosciences, AF3441, 1:1000) were used. To verify the effect of the JAK2/STAT4 pathway on proliferation, migration and gene expression of BMSC-CM-cultured fibroblasts, fibroblasts were treated with BMSC-CM or BMSC-CM + 10 μM AG490 (MedChemExpress, HY12000, USA). Then CCK-8 assay, wound-scratch assay and RT-PCR were performed following above-mentioned steps.

### Establishment of animal model

Vaginal distention (VD) was performed to simulate birth trauma-induced SUI in rats. All animal experiments were approved by the Animal Care Committee at First Affiliated Hospital of Wenzhou Medical University. Fifteen female Sprague–Dawley (SD) rats were equally divided into three groups: sham VD + control medium, VD + control medium, and VD + CCM. A 10F Foley catheter was inserted into the rat vagina and secured under anesthesia with 2% isoflurane. Next, a Foley balloon was inflated with 3 mL of saline to expand the vagina. After 4 h, the catheter was removed and 0.4 mL of control medium or CCM was injected around the urethra in the VD + control medium or VD + CCM group. The other rats were inserted with catheters, without simultaneous balloon inflation, and received an equal volume of control medium in the sham VD group.

### LPP measurement

LPP was measured to observe the degree of urinary incontinence. Two weeks after VD, the rats were anesthetized with 1.2 g/kg of urethane intraperitoneally, and the bladder was emptied before the tests. An epidural catheter was connected via a tee tube to a microsyringe pump and a pressure transducer, which was connected to a RM6240 signal acquisition system (Chengdu Instrument Factory, Chengdu, China). Next, the catheter was inserted into the bladder via the urethra, and the bladder was filled with saline at 10 mL/h via a syringe pump. When the capacity reached 0.5 mL, gradually increasing external pressure was applied with the finger to the bladder. During leakage, the external pressure was rapidly removed and the intravesical pressure that induced leakage was considered LPP. The test was repeated thrice in each rat and the mean LPP was calculated.

### Histology, IHC, and IF

After the LPP test, the rats were euthanized by an overdose of anesthesia. A catheter was inserted through the urethral orifice and the dome of the bladder to distinguish the urethra. Afterward, the middle urethra and AVW were carefully separated along the catheter. The tissues were fixed overnight in 4% paraformaldehyde, then dehydrated in an ascending alcohol gradient, followed by cleaning in xylene, embedding into paraffin, and cutting into 4-μm sections. The paraffin sections were successively heated, dewaxed, rehydrated, and prepared for collagen fiber staining, IHC or IF. The collagen fiber staining was performed using the modified Masson’s Trichrome Stain Kit (Solarbio, G1345, China) and following the manufacturer’s recommendations. For IHC, antigen retrieval was performed in sodium citrate solution, the endogenous peroxidase activity was inhibited with 3% hydrogen peroxide, and blocking was performed with 5% BSA solution. The tissue sections were subsequently incubated overnight at 4° C with an anti-collagen III antibody (1:100, Abcam, ab7778, USA). After washing with phosphate-buffered saline (PBS), an HRP-conjugated secondary anti-rabbit antibody was added for 1 h at 37° C. After washing with PBS, the sections were treated with 3,3′-diaminobenzidine (DAB) (Solarbio, DA1010, China). The reactions were stopped when brownish-yellow areas appeared. For IF, the above-mentioned steps were performed and an antibody against vimentin (1:200, CST, 5741S, USA) was used.

### Statistical analysis

The data are presented as mean ± standard deviation of the mean. All statistical analyses were performed using a GraphPad Prism 8.0.1. A two-sided Student’s *t*-test was used to analyze differences between the two groups. A one-way analysis of variance was used when more than two groups were analyzed. A *p*-value of less 0.05 was considered significant.

## Supplementary Material

Supplementary Table 1
