# Supplementary material for: Bone marrow stem cells secretome accelerates simulated birth trauma-induced stress urinary incontinence recovery in rats
Source: Aging (Albany NY). 2021 Mar 31;13(7):10517–34. doi: 10.18632/aging.202812 (PMC8064190; doi:10.18632/aging.202812)
Supplement: Supplementary Table 1 [file aging-13-202812-s001.pdf]

SUPPLEMENTARY TABLE

Supplementary Table 1. Potential transcription factors regulating DEGs.

DEGs	Fold change(CM vs. con)	Potential transcription factors (dissimilarity margin ≤ 5%)
POSTN	2.52	GR-beta C/EBPbeta HNF-1A STAT4 c-Ets-1 TFIID GR-alpha c-Ets-2 IRF-1 TFII-I NF-AT1 GATA-1 FOXP3 RXR-alpha C/EBPalpha LEF-1 HNF-3alpha PXR-1:RXR-alpha PR-B PR-A SRY HOXD9 HOXD10 AP-1 c-Jun HNF-1C HNF-1B GR Elk-1 NF-AT2 NF-AT1 STAT1beta TBP FOXO4 c-Myb YY1
COMP	2.14	YY1 C/EBPbeta GR-beta GATA-1 AP-2alphaA RXR-alpha TFIID HNF-3alpha HOXD9 HOXD10 GR-alpha WT1 I -KTS WT1 -KTS WT1 I WT1-del2 WT1 I-del2 Pax-5 p53 TFII-I STAT4 GCF ENKTF-1 Sp1 AP-1 c-Jun c-Fos C/EBPalpha
TGFBI	2.05	Pax-5 FOXP3 p53 C/EBPbeta E2F GR-beta XBP-1 GR-alpha C/EBPalpha YY1 RXR-alpha TFII-I AP-2alphaA c-Ets-2 ENKTF-1 Sp1 STAT4 HNF-1A c-Ets-1 Elk-1 NFI/CTF NF-1 TFIID GR PR-B PR-A ER-alpha MAZ
P4HA3	1.81	C/EBPbeta PEA3 TFII-I GATA-1 STAT4 c-Ets-1 GCF GR-beta YY1 GR-alpha PR-B PR-A RXR-alpha c-Ets-2 AP-2alphaA AP-1 c-Jun c-Fos Pax-5 p53 Sp1 Elk-1 T3R-beta1 NF-AT1 RAR-beta:RXR-alpha C/EBPalpha HNF-1A GR FOXP3 XBP-1
AKR1C3	1.46	GR-alpha GR-beta NF-AT1 GATA-1 TFII-I Pax-5 PR-B PR-A NF-AT2 STAT4 C/EBPbeta c-Ets-1 p53 c-Jun GR TFIID C/EBPalpha NF-Y FOXP3 YY1 c-Ets-2 AP-2alphaA NFI/CTF IRF-1 Elk-1 ER-alpha TBP c-Fos POU2F1 HNF-1A HNF-3alpha
LRRC15	1.62	GR-beta STAT4 c-Ets-1 C/EBPbeta TFIID HNF-1A FOXP3 HOXD9 HOXD10 HNF-3alpha GR-alpha TCF-4E GR YY1 c-Ets-2 IRF-1 NF-AT1 RXR-alpha RAR-beta AP-2alphaA TFII-I EBF PR-B PR-A Pax-5 p53 GATA-1 AP-1 c-Jun NF-1 NF-Y IRF-2 LEF-1
DDIT4	1.59	p53 STAT4 NF-kappaB RXR-alpha YY1 TFII-I GATA-1 C/EBPbeta TCF-4E GR-alpha AP-2alphaA GCF NF-kappaB1 c-Ets-1 Pax-5 Sp1 AR GR-beta TFIID NF-AT2 STAT1beta ENKTF-1 NF-Y C/EBPalpha NF-1 HIF-1 Sp3 RAR-beta:RXR-alpha RAR-beta NF-AT1 ER-alpha FOXP3 c-Myb
ATP1A2	1.59	E2F-1 C/EBPbeta TCF-4 c-Ets-1 GR-alpha GR-beta AR c-Ets-2 NF-Y GATA-1 RXR-alpha TFIID AP-2alphaA PEA3 AP-1 c-Jun FOXP3 c-Myb TFII-I Pax-5 p53 XBP-1 HNF-3alpha Sp1 YY1 STAT4 PR-B PR-A C/EBPalpha PXR-1:RXR-alpha ER-alpha
ANGPTL4	1.57	HIF-1 c-Myc USF1 RXR-alpha GR-beta YY1 C/EBPbeta AP-2alphaA RAR-beta ER-alpha Sp1 ETF GR-alpha p53 C/EBPalpha IRF-2 STAT4 c-Ets-1 Elk-1 GR TFIID XBP-1 c-Ets-2 RAR-beta:RXR-alpha E2F-1 AR TFII-I HNF-3alpha HOXD9 HOXD10 PITX2 Ik-1 USF2T3R-beta1 c-Jun
